# Neural population coding: combining insights from microscopic and mass signals

**DOI:** 10.1016/j.tics.2015.01.002

**Published:** 2015-03

**Authors:** Stefano Panzeri, Jakob H. Macke, Joachim Gross, Christoph Kayser

**Affiliations:** 1 Center for Neuroscience and Cognitive Systems, Istituto Italiano di Tecnologia, Corso Bettini 31, 38068 Rovereto, Italy; 2 Department of Physiology of Cognitive Processes, Max Planck Institute for Biological Cybernetics, Spemannstrasse 38, 72076 Tübingen, Germany; 3 Neural Computation and Behaviour Group, Max Planck Institute for Biological Cybernetics, Spemannstrasse 41, 72076 Tübingen, Germany; 4 Bernstein Center for Computational Neuroscience Tübingen, Germany; 5 Werner Reichardt Centre for Integrative Neuroscience Tübingen, Germany; 6 Institute of Neuroscience and Psychology, University of Glasgow, Glasgow G12 8QB, UK

**Keywords:** neural code, cross-correlation, relative timing, sensory processing, oscillation, multiscale processing, neuroimaging, state-dependent coding

## Abstract

Behavior relies on the distributed and coordinated activity of neural populations. Population activity can be measured using multi-neuron recordings and neuroimaging. Neural recordings reveal how the heterogeneity, sparseness, timing, and correlation of population activity shape information processing in local networks, whereas neuroimaging shows how long-range coupling and brain states impact on local activity and perception. To obtain an integrated perspective on neural information processing we need to combine knowledge from both levels of investigation. We review recent progress of how neural recordings, neuroimaging, and computational approaches begin to elucidate how interactions between local neural population activity and large-scale dynamics shape the structure and coding capacity of local information representations, make them state-dependent, and control distributed populations that collectively shape behavior.

## Highlights

• Neural population codes are organized at multiple spatial scales.
• Microscopic organization of neural codes reveals a key role of neural heterogeneity.
• Microscopic and population dynamics interact to make processing state-dependent.
• Additional computational analyses of neural activity across resolutions are needed.

## Neural population codes at multiple scales

In complex animals, information about behaviorally important variables such as sensory signals or motor actions is carried by the activity of populations of neurons [1–4]. Our understanding of neural information processing is founded on the conceptual assumption that, if two or more sensory stimuli can be discriminated, or two or more behavioral responses are different, their associated patterns of neural activity must be readily discriminable. Several key ingredients shape the capacity of a neural population code (see Glossary) to form such discriminable representations: the diversity of neural response properties, their spatial and temporal response profiles, the cross-neural correlations, and the state-dependence of cortical activity (Box 1).

Recent progress in understanding the contribution and the interplay of each of these key ingredients arises from both technical and conceptual developments. Experimental methods now allow measuring and manipulating up to hundreds of neurons simultaneously in behaving animals and permit a direct link between population codes and behavior [5–8]. Multi-scale studies combining invasive recordings with measurements of neuroimaging signals are becoming more frequent, allowing us to combine insights across methodologies [9,10]. Finally, advances in computational methods for single-trial analysis of multivariate data allow us to fully exploit the avenues opened by high-density brain measurements [11–14].

We review here how these developments facilitate the convergence of knowledge gained from invasive multi-neuron recordings, neuroimaging data, and mathematical modeling, and begin to reveal the organization and computations of neural population codes at multiple scales of organization. For simplicity, we mostly focus on the encoding of sensory variables, but the concepts are relevant for the generic encoding of any variable (for example, motor variables).

## The diverse response selectivity of sensory neurons

The computational properties of population codes are usually quantified using measures of the information they carry about sensory stimuli [15]. The most widely used quantifications of neural information are based upon Shannon information (quantifying the accuracy of discrimination among different stimuli in a set) or Fisher information (quantifying discrimination of small stimulus changes, or the accuracy of decoding an individual stimulus). Because most concepts reviewed here apply to both types of information, we only distinguish between them when necessary.

How a neural population represents information is partly determined by the diverse selectivity of individual neurons [16]. Individual neurons can carry information about sensory stimuli using a firing-rate code [17], for example by elevating their firing rate when presented with ‘preferred’ stimuli, and decreasing their rate when presented with other stimuli (Box 1). Nevertheless, even neighboring neurons may have heterogeneous stimulus selectivity [18,19]. For example, different neurons may have different stimulus tuning curves exhibiting a preference for different stimulus features or exhibiting different stimulus tuning widths. The heterogeneity of stimulus tuning generally implies that individual neurons may carry complementary information to that provided by others. As a result, the ability of a heterogeneous population to discriminate among stimuli in a set should, under most conditions, increase with population size. If individual neurons in a population each prefer well-separated stimulus features (such that their tuning curves do not overlap), the range of stimulus features encoded by that population would increase with population size. If, instead, neurons in a population have diverse but partly overlapping tuning curves, the complementary information carried by different neurons would lead to a better discrimination of stimulus features in the regions where the tuning curves overlap.

The scaling of information with population size depends on the structure of tuning preferences and of trial-to-trial response correlations, and its investigation can provide important insights. It can indicate how many neurons would be sufficient to achieve a desired level of sensory accuracy (assuming that the decoding mechanisms or later processing stages do not discard any of this information). Its extrapolation to infinite population size sets an upper bound on the information that can be achieved by a population with the considered response properties.

**Figure 1:**
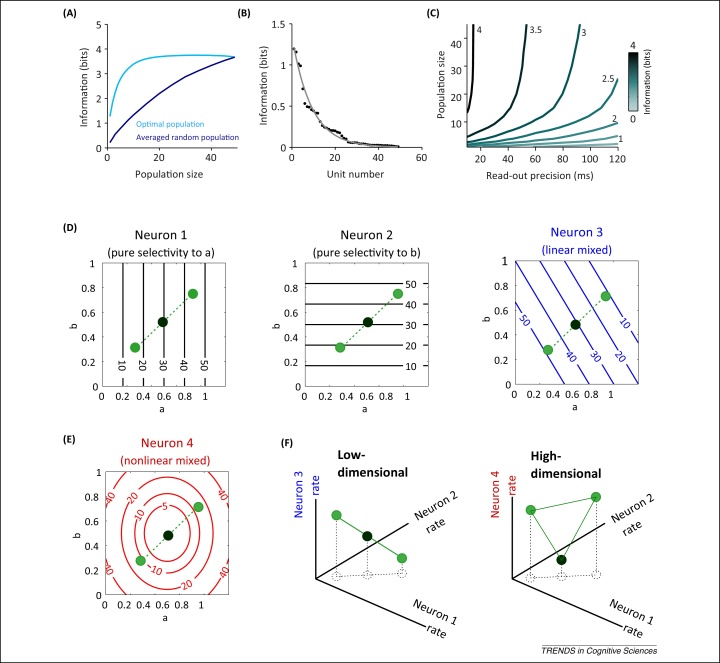
The impact of neural heterogeneity on population codes. Panels A–C plot information about natural sounds carried by neurons in primate auditory cortex and are reproduced with permission from [22]. **(A)** Stimulus information in randomly subsampled populations (dark blue) increases steadily with population size. However, an ‘optimized’ subpopulation built by selecting first the most informative neurons shows a much quicker saturation of information with population size (light blue), demonstrating that a small subset of neurons carries all the information available in the population. **(B)** The distribution of the information carried by single neurons (dots, with the line denoting a fit to an exponential distribution) shows a high heterogeneity: only a relatively small fraction of all recorded neurons carry substantial amounts of information. **(C)** Contour plot of stimulus information in optimized populations across variations in the read-out precision used for information decoding (*x* axis) and in population size (*y* axis), showing that the information that is lost when decoding responses at coarse temporal precision cannot be recovered by increasing population size. High values of information can only be reached with 5–10 ms precision, whatever the population size. Panels D–F: schematic of the importance of mixed nonlinear selectivity, reproduced with permission from [35]. **(D)** Responses (spikes/s) of hypothetical neurons to two continuous stimulus parameters (a,b) that characterize two stimulus features. Neurons 1 and 2 are tuned to a single parameter. Neuron 3 is a linear mixed-selectivity neuron whose response is a linear combination of responses to individual parameters. The circles indicate the responses to three sensory stimuli parameterized by three combinations of the two stimulus parameters. **(E)** Neuron 4 is a nonlinear mixed-selectivity neuron: its response cannot be explained by a linear superposition of responses to the individual parameters. **(F)** The population response projected onto two sub-spaces created by neurons 1,2,3 and 1,2,4, respectively (the axes indicate the firing rates of the neurons). The left case lacks nonlinear mixed-selectivity neurons, and hence the responses to the three stimuli lie on a line and cannot be discriminated by a linear classifier (an appropriately positioned plane). The right case includes a mixed-selectivity neuron, and thus the population responses to the three stimuli lie on a plane, making the stimulus discrimination possible with a linear classifier.

The scaling of information with population size is often studied by averaging information over subpopulations that are randomly sampled from the recorded neurons. This approach typically reveals a steady increase of information with population size (Figure 1A), and led to the hypothesis that increasing population size allows the encoding of arbitrarily high amounts of information [20,21]. However, recent work [22] has shown that this steady increase may actually be an artifact resulting from random subsampling. This artifact arises because of an often neglected aspect of neural heterogeneity [22]: only a small fraction of neurons in a given population carry significant sensory information in a specific context (Figure 1B). As a result of this heterogeneity in single-neuron properties, a small but highly informative subset of neurons is sufficient to carry essentially all the information present in the entire observed population (Figure 1A) [22].

The above results are part of an emerging picture that a small-dimensional subspace of the experimentally measured activity suffices to explain the population dynamics underling sensory processing [23] and motor behavior [24]. This picture is consistent with the observed sparseness of cortical activity [25] (at any moment only a small fraction of neurons are active) and is compatible with studies showing that perception and actions can be driven by small groups of neurons [26]. A sparse population code may be advantageous for metabolic efficiency and may facilitate dendritic computations requiring the separation of individual synaptic afferents [27,28].

Studies on the auditory system suggest that those neurons contributing the most to a population code are those that respond sparsely over time, and do so with precisely timed temporal spike patterns [22,29,30]. Importantly, the information carried by these precise spike patterns cannot be replaced by the information in the coarse-scale firing rates of other neurons in the population (Figure 1C), because information in spike patterns is complementary to that in firing rates and because the fraction of neurons carrying information by rates is limited [22]. This implies that spike timing remains crucial even for population codes [22,29,31]. The complementary nature of millisecond-scale spike patterns and firing rates has similarly been observed in the somatosensory [32] and visual system [33]. Thus, both the spatial and temporal dimensions are important for understanding neural population codes, raising questions as to how such precise timing or population sparseness can be assessed using neuroimaging.

## Benefits of mixed selectivity for population coding

In higher association regions the heterogeneity of cortical neurons expresses itself as patterns of selectivity to multiple sensory and task-related variables, which can be mixed in complex, sometimes nonlinear ways [34,35]. A pioneering study unveiled the advantages of nonlinear mixed selectivity [35]. If responses were selective only to individual task or stimulus aspects, or to their linear combinations (linear mixed selectivity; Figure 1D), the dimensionality of the neural representation would be lower than the number of neurons in the population. In the example in Figure 1D–F, the neural representation of a set of stimuli of two linearly mixed neurons lies on a line. This implies that complex nonlinear operations are required for decoding the information content (Figure 1F). By contrast, a heterogeneous nonlinear mixed representation (Figure 1E) leads to a richer population representation that has higher dimensionality than its linear counterpart and that can be more easily decoded by downstream areas using linear combinations of neural activity [36] (Figure 1F). In sum, heterogeneous nonlinear mixtures of selectivity increase the effective dimensionality of a population code and help to extract diverse information using simple linear decoding.

This high-dimensional population representation by mixed selectivity may seem at odds with reports of small subpopulations effectively encoding primary sensory information. However, sparseness and high dimensionality of mixed nonlinear selectivity can coexist and may even combine optimally under appropriate conditions [37]. In fact, when nonlinearly mixed neural representations are also sparse, they naturally achieve an optimal trade-off between the need to maximize the diversity of responses to different stimuli (increasing the stimulus discrimination properties of population responses), and the need to maintain high response reliability (i.e., achieving consistent responses for noisy variations of the input) [37]. Nonetheless, systematic studies comparing the sparseness of neural responses across brain regions will be necessary to clarify the extent to which neural populations take advantage of the potential benefits offered by sparse representations. Neuroimaging appears suited for this goal because it allows comparing neural representation spaces across species and sensory systems, and tracing these over time during task performance [9,14,38].

## Correlations of spike rates between neurons

The computational properties of a population code depend also on the pattern of response correlations between neurons [39]. Traditionally, a distinction is made between signal correlations (quantifying the correlation of the trial-averaged neural responses across the different stimuli, and thus quantifying the similarity of stimulus tuning) and noise correlations (quantifying the correlation of trial-by-trial variations of response at fixed stimulus attributes). Much work on population codes has focused on correlations between the firing rates of pairs of neurons.

When tools for simultaneous multi-neuron recordings became widely available in the 1990s, two divergent views were proposed about the impact of noise correlations between firing rates. The first holds that noise correlations of firing rates – if modulated by the stimulus – may act as a separate coding mechanism complementary to firing rates [40,41]. For example, it has been proposed that the dynamic stimulus-dependence of noise correlations among visual neurons (originating from changes in gamma-band synchronization among populations) carries information about whether individual neurons respond to the same or separate objects [42]. This view has deeply influenced the interpretation of neural mass signals measured by neuroimaging because it suggests that changes in correlation (or coherence) between the activities of distinct populations may reflect how these populations encode information. The second view is that noise correlations of firing rates may impose limits on the growth of information with population size [43]. For example, for neurons with identical tuning curves, the presence even of weak positive noise correlations precludes the possibility to improve the accuracy of sensory information encoding by simply averaging the rate of many neurons (correlated noise can only be removed to a limited extent by averaging). This second view influenced studies on neural population codes, and motivated recent work on how neuronal networks may dynamically decrease noise correlations to reduce their potentially limiting effect on information encoding [44,45]. It has also been argued that the limiting effect of correlation may partially be overcome in populations with heterogeneous tuning and correlation properties [46,47].

The potential impact of correlations on population coding has been studied extensively using model-based approaches that quantify stimulus information under various assumptions about correlation structures or readout mechanisms [15,48,49]. This approach has shown that correlations can influence neural population coding in more complex way than described above: depending on the precise pattern of signal and noise correlations, and the choice of readout mechanisms, correlations can increase, decrease, or leave unaffected the information carried by a population [1,15,40,49–51]. An important insight of these theoretical studies is that the precise pattern of noise correlation matters more than noise correlation strength or population size. For example, a recent study [49] considered the ability (quantified using Fisher information) to estimate small changes in a stimulus from population activity, and found that the type of noise correlations that limit the increase of information with population size are so-called differential correlations. Intuitively, such differential correlations correspond to correlated trial-to-trial variability of neural responses that shift the profile of population activity such that it looks like the population activity that would have been elicited by a slightly different stimulus. Thus, this correlated variability makes a noisy fluctuation of population activity look like the signal representing a different stimulus value, and thus acts as a source of noise that cannot be eliminated even by increasing the number of neurons. Whether these information-limiting correlations actually occur can be empirically investigated by computing information versus the number of neurons and investigating whether information saturates with population size. Because information depends crucially on the structure of correlations, extrapolation of its dependence on population size from real data should be performed using decoding approaches [16,49] rather than making strong but difficult-to-validate assumptions about the structure and shape of correlations [49].

Besides determining the amount of information carried by neural populations, noise correlations in firing rates may also be indicative of important computational functions [52]. Noise-correlations may have a role in probabilistic codes representing sensory uncertainty by reflecting correlations in the uncertainty associated with individual stimulus variables [53,54]. For example, for an ambiguous input consistent with one of two possible stimuli, the neurons representing these two stimuli would be negatively correlated because evidence for one stimulus speaks against the other [55]. It has also been argued that positive correlations within larger populations make the code sparser by increasing periods of population quiescence, and hence concentrate information into rare periods of common activity [56–58]. This latter property may strongly shape how cortical activity is seen through neuroimaging.

## Correlations of spike timing between neurons

Correlations between spike times can also contribute to population codes, for example by facilitating the readout of temporal response patterns in a relative latency code. Individual neurons can encode stimuli by their response latency (that is, the delay between stimulus onset and neural firing) [59,60]. Although information in the latency of an individual neuron may not be directly accessible to a downstream decoder (because measuring latency requires information about the precise time of stimulus onset), information in the relative latency between neurons may be robustly extracted by exploiting the fact that trial-by-trial shifts of latency are often correlated across a population [61–63]. For example, latencies of different neurons may tend to be all-late or all-early because of global fluctuations in network excitability. Correlated latency shifts preserve the relative order of firing or latency differences across neurons, whereas uncorrelated shifts do not. In auditory cortex, the readout of such relative timing may be further supported by time-reference events, in other words, population response patterns that encode the stimulus time with an early, stimulus-invariant latency [64]. Stimulus-selective neurons can then encode the stimulus identity by the time of their spikes relative to these reference events [64]. Although these relative-latency codes are often studied at the pairwise level, recent work suggests that they may reflect a more general larger-scale organization of population activity: groups of neurons may be co-active in stereotyped sequences (termed packets [65]), and the relative timing and strength of each neuron within this sequence may encode specific stimulus attributes [65,66].

Finally, correlations between spike times may also ensure the transmission of information across areas, for example by facilitating the impact on post-synaptic targets [67,68] or by facilitating the selective read-out of specific combinations of afferents [69,70]. All in all, this suggests that the relative timing between neurons contributes both to representing sensory information and to transmitting this information between areas.

## State-dependence of population codes

Local neural activity not only depends on the current sensory input but also on the current brain state [71,72]. Although feed-forward inputs to local circuits provide sensory afferents, the abundance of recurrent and feedback connections, and of neuromodulatory inputs, shapes the background activity on which this sensory information is processed [73–75]. For example, neuromodulatory inputs that are not directly stimulus-related (such as cholinergic or noradrenergic signals that are an essential component of the control of the animal's behavioral state) can profoundly change the excitability of sensory cortical circuits, the degree of correlation among neurons, and the gain and reliability of individual neurons [76,77]. Variations in brain state account for a significant fraction of the trial-to-trial variability of population activity [78].

This state-dependence must have profound – but largely unexplored – implications for how population codes operate. State-dependence may imply that populations transmit information only using codes that are robust to state fluctuations (e.g., relative latencies or relative firing-rates). Alternatively, downstream areas may extract variables indicating the current state from network activity (similarly to procedures used in data analysis [78]) and then use state-dependent decoders to interpret population activity. Progress in understanding state-dependent coding requires better statistical methods for single-trial analysis that can be applied to different measurements of brain activity to disentangle state-dependent and -independent aspects of a code [12,79–81].

## Insights on population coding from mass activity

Although a complete description of population codes may require recording all neurons involved in the considered task, important empirical knowledge about population codes can be gained from measurements of neural mass signals with high temporal resolution (LFP, ECOG, EEG, MEG). These measurements lack cellular-level resolution but can be easily applied to multiple brain areas and complex tasks, and are sensitive to both supra- and sub-threshold activity. Importantly, they have the potential to capture indicators of cortical state that cannot be easily extracted from the spiking activity of a few neurons alone [11].

Advances in single-trial data analysis have expanded the use of mass signals to study sensory transformations, permitting researchers to study the same questions using neuroimaging, multi-neuron recordings, and computational models. For example, recent neuroimaging studies extracted features of population activity influencing the variability of single-trial percepts [82–84], and demonstrated that visual object categories [85] or fine details of auditory signals [86–90] can be recovered using stimulus reconstruction or decoding methods.

**Figure 2:**
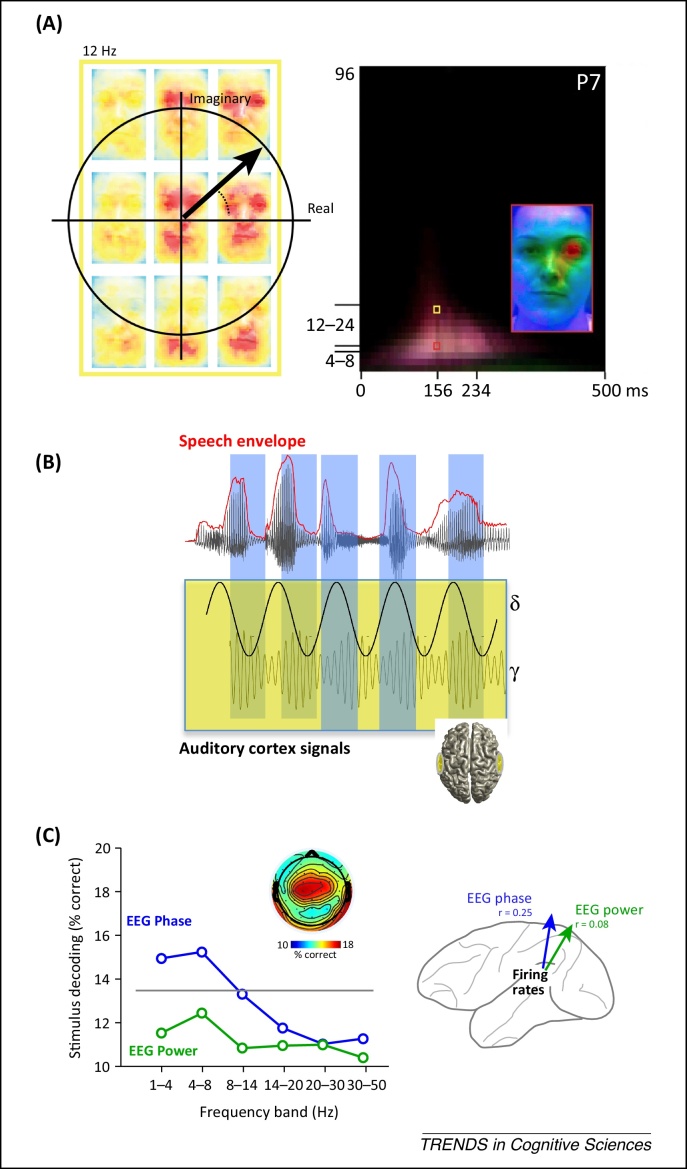
Phase coding observed from single-trial analysis of mass signals. **(A)** Encoding of visual features in the phase of EEG signals. (Left) Information about visual stimuli carried by time-frequency EEG data reveals phase-specific coding of stimulus features (eyes and mouth of a face). (Right) Time-frequency representation of feature coding. In both panels, information carried by the EEG signal is color-coded, with warmer colors indicating higher information values. The contralateral eye was encoded by the phase at the 10 Hz signal (reproduced from [94]). **(B)** Phase encoding of continuous speech in auditory cortex. Theta-band (3–7 Hz) phase in bilateral auditory cortex dynamically encodes temporal variations in the envelope of continuous speech and modulates the amplitude of high-frequency gamma oscillations (reproduced from [92]). **(C)** Correlation between the performance (quantified as percentage of correctly decoded trials) in decoding which natural sound was presented when using auditory cortical firing rates in non-human primates, and the performance in decoding natural sounds when using theta-band EEG phase/power in humans. The same natural sound stimuli were presented to both species. Theta-band EEG phase captures better the stimulus selectivity of cortical firing rates (reproduced from [91] with permission of Oxford University Press).

Additional insights can be gained from mass signals by decomposing them into specific frequency bands and separating the sensory information carried by power and phase of individual bands. Recent work, based on both invasive (LFP) and non-invasive (EEG and MEG) recordings, has individuated the phase of low-frequency activity as a particularly informative feature of mass activity [85,91–95]. In the visual system, detailed sensory features are reflected more in the phase of low-frequency (below 12 Hz) activity than in the power [1,94,96] (Figure 2A). Related findings were made in the auditory system, where the phase of low-frequency activity encodes speech or complex sounds [91–93,95,97] (Figure 2B,C). Stepping beyond single-site analysis, some studies found that the relative timing of neural responses across different sites carries more sensory information than the activation of individual sites [85,98,99]. These results hence suggest that the relative timing of population activity could at least be as important for sensory coding as the strength of activity of an individual population, in line with insights from studies on spiking activity that highlight the role of relative response timing.

An important population coding principle emerging from the analysis of mass signals (both using MEG and EEG [92,94] or intracranial recordings [60,100,101]) is that of multiplexing of sensory information: different frequency bands of population activity each carry complementary information about stimulus features. For example, nested patterns of slower (e.g., delta or theta) and faster (e.g., gamma) auditory cortical rhythmic activity encode complementary aspects of speech (Figure 2B) [92]. These results illustrate the multiple coding dimensions of population signals, and highlight the importance of understanding how specific aspects of coding in spiking activity map onto phase and power of rhythmic mass signals. Specific challenges for understanding the link between non-invasive neuroimaging and neural population codes are outlined in Box 2 and in the outstanding questions (Box 3).

## Insights from the combined observations of single neurons and mass signals

Important insights about population codes further arise from studies that simultaneously measure spiking responses and mass signals, or that perform comparative analysis on such data obtained in separate experiments.

One key opportunity of the joint measurements arises from the complementary nature of single-neuron firing and of mass signals: mass signals capture aspects of sub-threshold activity and of intrinsically driven state-changes that cannot be measured by observing the spiking activity of a few neurons alone [11,102]. Thus, low-frequency mass activity can be seen as a measure of the background state fluctuations constituting the ‘context’ that affects processing of the ‘content’ carried by sensory inputs [103]. Examining the responses of individual neurons relative to the phase of low-frequency network rhythms can shed light on how the local circuit context affects the specific sensory content encoded in spiking activity.

**Figure 3:**
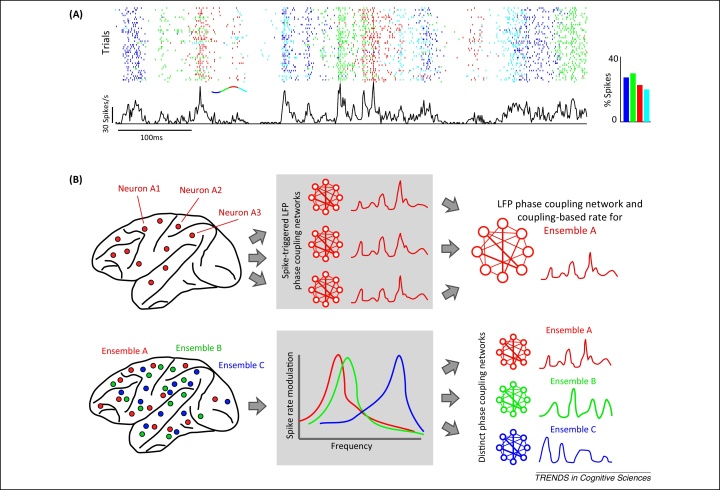
Insights about population coding from joint analysis of spiking activity and mass signals. **(A)** State-dependent neural firing in auditory cortex (data from [101]). The raster displays the response of one auditory cortical neuron to several repeats of a sequence of naturalistic sounds. Each spike is colored with the phase of the 4–8 Hz local field potential (LFP) (see right-hand inset for the color-coding of phase quadrants). This neuron is more active during the blue and green phase periods (as shown by the histogram of spikes at different phase quadrants, right inset), suggesting that LFP phase indexes changes in network excitability. Note that at several points during stimulus presentations there are identical firing-rate peaks that can be discriminated between each other only by the different phase ‘color’ at which they are fired. This means that the phase adds information complementary to that of spikes. **(B)** Schematic of how patterns of oscillatory phase coupling across multiple brain areas may coordinate anatomically dispersed neuronal ensembles (reproduced, with permission, from [106]). (Top row) How patterns of phase coupling among many different areas may synchronize anatomically dispersed neuronal ensembles. Electrodes at several locations in the monkey brain (left) identify that neurons fire preferentially in the presence of specific patterns of phases across different recording sites (indicated by network diagrams in center and right panels). Neurons that lock to the same or similar patterns of phases show the same time course of firing (center). This makes it possible to identify a pattern of LFP–LFP phase coupling across areas (center) that recruits a cell ensemble A (right) comprising the neurons A1, A2, and A3. (Bottom row) How the differential sensitivity to distinct brain rhythms or coupling patterns permits selective control of multiple coactive ensembles. (Left) Multiple functional ensembles, each spanning several brain areas, overlap in space. (Center) Interference between ensembles is minimized when each ensemble responds to a different frequency (ensembles A and C) or distinct phase-coupling pattern (ensembles A and B). (Right) Frequency and pattern selectivity permits dynamic, independent coordination of multiple coactive ensembles.

Converging studies suggest that single-neuron spike timing depends on the frequency-specific oscillatory LFP phase (Figure 3A), and that the sensory information carried by spiking activity can be better interpreted when the network context during which spikes were emitted is known [101,104] (Figure 3A). A recent study in the human medial temporal lobe showed that perception-related spiking activity is locked to an early stereotyped increase in theta LFP signals, suggesting that specific interactions between network rhythms and firing of individual neurons may define temporal windows facilitating conscious perception [105]. Complementing measurements of spiking activity with LFP recordings may allow differentiating otherwise ambiguous population states and provides a means to disentangle context-related activity fluctuations from those induced by sensory noise or other causes.

Joint measurements of single-neuron spikes and mass signals also facilitate our understanding of the coordination of population codes across brain structures. Recent concurrent multi-site recordings of spiking activity and LFPs have shown that the spiking activity of a single neuron depends not only on the phase of the LFP oscillation at the same site but also on the dynamic patterns of the relationships (or ‘phase coupling’) between the phases of neural oscillations measured by LFP at multiple distant sites [106] (Figure 3B). The similarity of preferences in LFP phase coupling between neurons also predicts the similarity of their firing-rate variations: neurons that prefer similar patterns of phase coupling exhibit similar changes in firing rates with time or task, whereas neurons with different phase preferences show divergent firing-rate dynamics [106]. This provides direct evidence that large-scale functional connectivity shapes local activation patterns and controls the co-activation and coordination of anatomically dispersed but functionally integrated ensembles of neurons, thus breaking new ground in the understanding of the large-scale organization of neural population codes.

Another approach to clarify the neural basis of the sensory information carried by different components of MEG/EEG signals is based on the use of information theoretic or stimulus-decoding methods [16] to compare quantitatively the similarities of stimulus encoding in mass signals and multi-neuron recordings [9,14,38]. This approach revealed (Figure 2C) that the phase (and to a much lesser extent the power) of low-frequency mass activity captures some aspects of the sensory information carried by population spiking activity [91]. Such comparative approaches will be crucial for understanding what mass signals can tell us about the underlying neural information processing (Box 2) and open the possibility to study emerging principles such as multiplexing, mixed selectivity, or of state-dependence across scales of brain measurements.

## Concluding remarks

Understanding the properties and principles of cortical population codes requires identifying the most informative patterns within the spatio-temporal complexity of multi-neuron activity and understanding how coding properties are affected by large-scale state changes. Invasive recordings provide detailed access to the heterogeneity, temporal precision, and correlation structure of multi-neuron activity, properties which recent computational studies highlight as being crucial for shaping the information-coding properties of a population. Mass signals, on the other hand, provide more direct access to large-scale changes in network state and connectivity, other crucial properties that shape information coding and which can account for trial-by-trial variations in cognitive tasks. Combining insights from both mass signals and multi-neuron recordings is a key challenge for the future.

In light of the reviewed properties of population codes several questions emerge about the integration of microscopic and macroscopic structure of population codes (Box 3). For example, can the mechanisms that seem crucial at the microscopic scale (such as neuronal heterogeneity, sparseness, or correlations or mixed selectivity) be observed from mass signals? In turn, how do specific patterns of activity observed at the macroscopic scale (such as patterns of phase coupling across multiple areas or global state changes) relate to the coding properties that are crucial at the microscopic scale? For sure, much is to be learned by methods, such as single-trial stimulus decoding or reconstruction methods, that allow a comparative and detailed assessment of similarity and complementarity of activity at each spatial and temporal scale.

To enhance our understanding of population coding, future developments of methods for high-dimensional data analysis and neural modeling will be necessary as well. Recent work has provided improvements in analytical techniques for reducing high-dimensional datasets and extracting the relevant sensory representations [11–14]. In addition, biophysical models of sensory representations in cortical microcircuits allow a principled and rigorous direct link between neural signals and computing architectures, facilitating our understanding of how coding principles are implemented in the neural circuits. Being able to subject different neural signals to the same scientific question and analysis routine, and being able to compute both spiking and mass signals from the same plausible neural network models [11], are two crucial features to further improve our understanding of neural population coding in the future.

## Figures and Tables

**Figure I:**
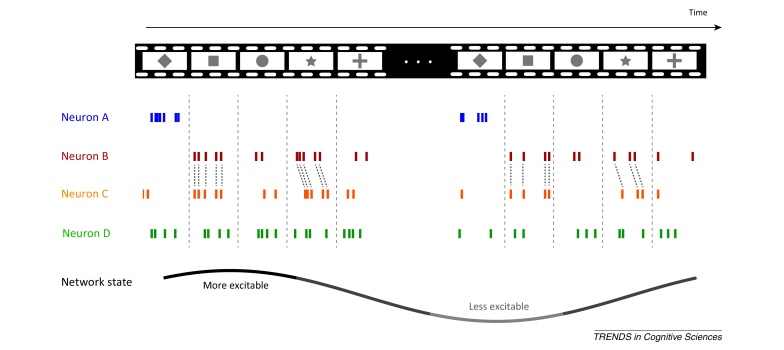
Schematic illustration of informative neural response features in a population code. The figure illustrates the responses of four neurons (A–D) to a set of five different stimuli each repeated twice (the geometric forms in the frames of the top row). Each small vertical line denotes one action potential. The slow wave at the bottom denotes a mass signal (e.g., an LFP) capturing the different phases of ongoing slow fluctuations in network state or excitability.

## Box 2 Comparisons of stimulus selectivity in mass signals and spiking activity

Measurements of neural mass activity with high temporal resolution (LFP, MEG, EEG, ECoG) provide rich signals varying on multiple timescales [110] and exhibiting reasonable spatial specificity [110]. Nevertheless, owing to the multiple neural phenomena they capture, and because of uncertainties in source localization and to cancellation of local signals [11,102,108], they are difficult to interpret in terms of the underlying neural processes. However, some relations between mass signals, spiking activity, and sensory encoding are emerging from recent work.

Specific frequency components extracted from MEG and EEG signals seem to be particularly predictive of neural firing rates. Combined neural and EEG recordings from visual cortex show that the phase of low frequency (<10 Hz) and the amplitude of high-frequency (gamma, 40–100 Hz) rhythms carry the most information about the strength of local firing, and each offer complementary information about the firing rate [111]. An indirect comparison of auditory stimulus selectivity in firing rates and EEGs revealed the strongest association with the phase (but not power) of low-frequency components [91]. How do the phase of low-frequency components, and the amplitude of faster components, relate to sensory encoding?

The phase of low-frequency (<12 Hz) rhythms likely reflects changes in local network excitability that shape both stimulus responses and spontaneous background activity. Evidence for this comes from neural recordings [112] and from neuroimaging studies demonstrating a causal relation between the phase of low-frequency activity and single-trial stimulus detection [113–115]. Hence, the phase of low-frequency network activity may indicate whether or not local networks are in a state facilitating the encoding of sensory information and driving perception. Some theories suggest that the excitability fluctuations marked by the low-frequency phase help to prioritize the encoding of salient features [60,115].

Does this phase also indicate what is encoded? Recent work suggests this is the case: low-frequency phase can reflect the slow temporal structure of dynamic sensory stimuli. Experimental data and network models show that slow brain rhythms entrain to low-frequency variations of natural stimuli such as speech, directly inducing sensory information in the phase of these signals (e.g., [91,92,116,117]).

In visual cortices the amplitude of gamma rhythms is the signal component carrying the most sensory information [118]. Models of gamma generation suggest that its amplitude reflects the instantaneous strength of local interactions between inhibitory and excitatory neurons, which is roughly proportional to the stimulus input – if excitation and inhibition are balanced [119]. Thus, gamma amplitude is expected to encode aspects of current stimulus features rather than the slow stimulus dynamics (which is reflected by slower signal components). The validity of this hypothesis requires further experimental tests, as does the causal role of such rhythms for behavior.

## Box 3 Outstanding questions

• How is it possible that mass signals carry sensory information given that only a small fraction of neurons are informative? How does neural heterogeneity affect what we can learn from mass signals?
• How do microscopic-level neural coding mechanisms (neuronal heterogeneity, sparseness) interact with macroscopic scale aspects of neural activity (patterns of coupling across multiple areas or global state changes)?
• Is mixed selectivity a hallmark of higher (association) brain regions, and does the mixed selectivity (previously observed across neurons) also exist in the time or frequency domains of neural activity (i.e., do different temporal signal components exhibit mixed selectivity)?
• What do neural correlations imply for mass signals, and how can we infer some properties of the microscopic structure of neural population codes from mass signals?
• How do local descriptions of cortical state (derived from multi-neuron recordings) relate to state-dependent signatures visible in neural mass activity?
• How can we best integrate recent advances in cellular imaging and histochemical or morphological analysis to disentangle the contribution of specific cell types to neural population codes?

## Glossary

Brain state: brain activity dynamics on a timescale of seconds. It reflects the interactions between ongoing endogenous activity and sensorimotor processing, and is strongly influenced by neuromodulatory systems.
Complementary information: two neural codes carry complementary information if the information they carry jointly is higher than the information carried by either code individually; this implies that some information not available in one code is provided by the other.
Decoding: the estimation (by either the brain or an artificial decoder) of a variable of interest (for example, the value of a presented sensory stimulus, or a motor variable) from the observation of a single-trial neural population response.
Differential correlation: a noise correlation between the firing rates of a pair of neurons whose strength, for each stimulus value, is proportional to the product of the derivatives of the tuning curves.
Dimensionality of the neural representation: the minimal number of coordinate axes that are needed to describe the variations of responses across all trials to all different experimental conditions (for example, all combinations of sensory stimuli and behavioral responses).
ECoG: electrocorticography.
EEG: electroencephalography.
Encoding: the generation of the set of specific activity patterns that represent a sensory attribute.
Firing-rate code: neural code that represents stimulus attributes using the number of spikes emitted in response to the stimulus, regardless of their temporal pattern.
Fisher Information: a measure of the variance of estimation of a particular stimulus value (e.g., contrast of a grating) from the single-trial observation of neural population activity.
Information: a measure of how much knowledge about which stimulus is being presented can be gained from a single-trial neural population response. Information is often quantified as Shannon Information or Fisher Information.
Latency code: a specific form of neural code encoding information in the timing of the response onset. The time of the response onset is usually measured with respect to stimulus presentation time, but can be defined also relative to another neural event (relative latency code).
Local field potential (LFP): a neurophysiological signal obtained by low-pass filtering extracellular recordings. It captures slow components of both sub- and supra-threshold neural activity.
Mass signal: a signal that comprises the aggregate neural electric activity in a local region and captures both supra- and sub-threshold phenomena, including spiking and synaptic activity. Examples are LFPs, ECOG, MEG, and EEG.
MEG: magnetoencephalography.
Multiplexing of sensory information: neural coding scheme in which complementary information is represented in different frequency components or temporal scales of neural population activity. For example, when different information is represented by the precise timing of individual spikes on the scale of milliseconds and by the slow modulation of the spike count on the scale of hundreds of milliseconds.
Neural population code: the set of response features of a population of neurons that carry all information about the considered stimuli. These features consist of spatio-temporal sequences of action potentials distributed across neurons and/or time.
Noise correlation: a measure of correlation between the firing of a pair of neurons that cannot possibly be attributed to the sensory stimulus. Noise correlation is quantified as the correlation between the firing of the neurons in response to a fixed external stimulus.
Packet: a pattern of firing consisting of a group of neurons firing transiently in a relatively stereotyped sequence. A packet may encode different stimulus features by modulating the relative timing and relative firing rate of the subsets of neurons firing within the sequence.
Phase/power: the current period within a cycle of a given oscillation / the amplitude of an oscillatory signal.
Readout mechanisms: a set of biophysical computational mechanisms used by the brain to extract information out of a neural population response.
Shannon Information: a measure of how much (on average) observation of a neural population response reduces the uncertainty about which stimulus (among those in a set) is being presented. Also called ‘mutual information’.
Signal correlation: a measure of correlations between the firing of a pair of neurons that are attributable to the sensory stimulus. Signal correlations are quantified as the correlation of tuning of the firing of the pair of neurons to the stimuli (for example, the correlation across stimuli of the tuning curves).
Temporal spike pattern: a repeatable temporal sequence of spikes that carries information about stimuli.
Time-reference events: a neural (single-neuron or population) activity pattern that encodes the time of an important event (for example, the onset of a stimulus) by emitting a transient response with a stereotyped, invariant, and short latency.
Tuning curve: a mathematical function describing the dependence of the trial-averaged firing rate of a neuron upon the value of the stimulus.
Tuning width: a measure of the selectivity of neural firing to stimuli. In intuitive terms, a narrow (respectively coarse) tuning width means that only few (respectively many) stimuli elicit a strong neural response.
